# Genetic and phylogenetic analyses of the first GIII.2 bovine norovirus in China

**DOI:** 10.1186/s12917-019-2060-0

**Published:** 2019-09-02

**Authors:** Zhihai Shi, Wenjia Wang, Zhaoxue Xu, Xiaozhan Zhang, Yali Lan

**Affiliations:** 1 Institute of Animal Husbandry and Veterinary Science, Henan Academy of Agricultural Sciences, Zhengzhou, 450002 Henan China; 2 Henan Key Laboratory of Farm Animal Breeding and Nutritional Regulation, Zhengzhou, 450002 Henan China; 3 College of Pharmaceutical Engineering, Henan University of Animal Husbandry and Economy, Zhengzhou, 450046 Henan China; 4 College of Veterinary Medicine, Henan University of Animal Husbandry and Economy, Zhengzhou, 450046 Henan China

**Keywords:** Bovine norovirus, Diarrhoea, Phylogenetic analyses, China

## Abstract

**Background:**

Norovirus (NoV) is recognized as a highly contagious enteric pathogen of mammals, and bovine norovirus (BNoV) is associated with calf diarrhoea and has caused great economic losses in the cattle industry.

**Results:**

Here, we describe a case of emerging calf diarrhoea on a cattle farm in Henan Province, Central China. BNoV was the only enteric pathogen detected in outbreaks according to tests for enteric viruses, bacteria and parasites. The complete genome of the newly identified strain CH-HNSC-2018 was successfully sequenced and found to be 7342 nucleotides in length. Sequence and phylogenetic analyses revealed that CH-HNSC-2018 belongs to GIII.2 BNoV. Further analysis of the major capsid protein demonstrated that it is separated by specific genetic distances from previous BNoV strains identified in China and has 4 new amino acid (aa) mutations, 134A, 327 T, 380 L and 423A, in the VP1 protein and 11 aa substitutions in the hypervariable P2 subdomain, suggesting that the BNoV strains circulating in China are diverse.

**Conclusions:**

This is the first detection of GIII.2 BNoV in the VP1 region in China. This report should form a basis for further molecular studies on NoV and bovine enteric viruses in China.

**Electronic supplementary material:**

The online version of this article (10.1186/s12917-019-2060-0) contains supplementary material, which is available to authorized users.

## Background

Norovirus (NoV) is an emerged non-enveloped virus with a positive-stranded RNA genome that widely infects humans and other animals and causes severe gastrointestinal disorders and even fatal bleeding [1]. NoV belongs to the family *Caliciviridae* and genus *Norovirus*, and its genome is approximately 7.5–7.7 kb, which encodes for 3 open reading frames (ORF1–3). ORF1 encodes a large polyprotein that is cleaved into 6 nonstructural proteins (N-terminal-NTPase-3A-like-VPg-3C-like-polymerase); ORF2 encodes VP1, the major capsid protein; and ORF3 encodes VP2, a minor structural protein. The pathogenesis of calicivirus remains unclear, and little is known about NoV. One important reason for this lack of knowledge is that NoV has rarely been successfully propagated in cell culture; the sole exception is the culture of a murine isolate.

NoVs are classified into seven genogroups (GI-GVII). Bovine norovirus (BNoV), which belongs to GIII (NoVs-GIII), is divided into two distinct genotypes based on amino acid (aa) diversity of the complete VP1 sequence, genotypes 1 (Bo/Jena/1980/DE) and 2 (Bo/Newbury2/1976/UK), which were originally discovered in Germany [2] and England [3], respectively. Recently, a dual nomenclature system was established to characterize the genomic properties of BNoV strains based on the sequences of RNA-dependent RNA polymerase RdRp (end of ORF1) and major capsid protein (beginning of ORF2). Based on this novel nomenclature system, BNoV GIII is reclassified into GIII.P1_GIII.1 (which contains polymerase and VP1 genes from the Jena strain), GIII.P2_GIII.2 (which contains polymerase and VP1 genes from the Newbury2 strain), GIII.P1_GIII.2 (which contains a polymerase gene from the Jena strain and a VP1 from the Newbury2 (recombinant) strain, e.g., the Bo/Thirsk10/00/UK strain), and GIII.P2_GIII.1 (which contains a polymerase gene from the Newbury2 strain and a VP1 gene from the Jena (recombinant) strain, e.g., the B-1SVD/03/US strain) [4]. Moreover, some studies have employed ORF3 to characterize the phylogenetic topology of BNoV [5]. Until now, the available BNoV strain sequences have been limited [6] and have not provided enough data to establish intensive phylogenetic relationships or rates of evolution of BNoVs. To date, BNoV has been reported worldwide, including in China [7], Iran [8], Egypt [9], Argentina [10], Italy [11], the USA [12], and New Zealand [13], leading to substantial loss to the worldwide bovine industry. Furthermore, serum antibodies against BNoV (GIII) have been detected in humans [14], and sequences of human NoVs have been identified in bovine stool specimens [15], suggesting possible interspecies transmission [16].

Although BNoV plays a role in the aetiology of calf enteritis [17], data on emerging enteric viruses in cattle in China are scarce. Until now, only one report, from 2018, has reported BNoV from calves aged 3 to 4 months, which had a low BNoV GIII.1 infection rate (3/28) in diarrhoea faecal samples from Hebei and Sichuan Provinces, China 7. Here, we detected a newly emerging BNoV strain from a cow farm in Central China, sequenced its whole genome and analysed its phylogeny and mutations. The newly identified strain was classified as genotype 2 and was distantly related to the previously identified Chinese BNoV strains. This study systematically describes the genetic and phylogenetic characteristics of the newly identified BNoV strain and highlights that continuous surveillance is needed to help develop suitable vaccines and reasonable control measures.

## Results

### Detection of the BNoV

To determine the causative pathogen of the outbreak on the studied cattle farm in Henan Province, Central China, RT-PCR was used to detect the common potential pathogens causing calf diarrhoea. The diarrhoeal faecal samples were positive for BNoV (2/8, 25%), whereas no other viruses were detected, including BRV (A, B, C), BCoV, BVDV, BKV, BAstV, BNebV, or BToV. The samples were further cultured in MDBK cells to isolate the causative agent; no cytopathic effect was observed, and no BNoV nucleic acids were detected in the fifth blind passage. The newly detected viral strain was named CH-HNSC-2018. The results demonstrate that BNoV is an emerging pathogen of calf diarrhoea in this outbreak.

### Sequencing the BNoV viral genome

To our knowledge, although the virus was first detected in China in 2018, this study is the first to report the complete genome of GIII.2 and its phylogenetic characteristics in China. CH-HNSC-2018 genomic RNA consists of 7342 nucleotides (nt), with a 19-nt poly (A) tail. The BNoV genome contains three sequential ORFs starting from the 22nd nt: ORF1, 5055 (22–5076) nt; ORF2, 1569 (5063–6631) nt; and ORF3, 651 (6621–7271) nt, which encode a nonstructural polyprotein and the structural VP1 and VP2 proteins, respectively. ORF1, ORF2 and ORF3 overlapped each other by 14 and 10 nt, respectively. The 5′ untranslated region of our strain has as many as 21 nt, similar to Newbury2 (21 nt) and Jena (21 nt) strains. The G + C content of our strain genome was 57.18%, similar to that of the Newbury2 (57%) [18] and Jena (56%) [19] strains. The complete sequence was deposited in the GenBank database, and the accession number is MN122335.

Consistent with other BNoVs, the protease cleavage sites of CH-HNSC-2018 were LQ/GP, LQ/AP, VQ/AP and LE/GG (Additional file 2: Figure S1). Meanwhile, aa motifs typical of caliciviruses were also observed in the CH-HNSC-2018 ORF1 polyprotein in NTPase (^490^GPPGIGKT^497^), 3C protease (^1134^GDCG^1137^), and RNA-dependent RNA polymerase (RdRp) (^1473^GLPSG^1477^ and ^1517^YGDD^1520^), as previously described for the Newbury2 and Jena strains.

### Homology and phylogenetic analysis of BNoV

In this study, we further analysed the BNoV gene to characterize the enteric pathogen. Nucleotide sequences analysis indicated that CH-HNSC-2018 was closely related to BET-17 (MK159169, CHN) with 87.6% nt identity. Further analysis revealed that the ORF1 gene of BNoV was the most similar region between CH-HNSC-2018 and BET-17, with greater than 95.4% nt and 97.3% aa identity (Table 1). However, the major capsid protein (VP1) was closer to CV186-OH (AF542084, USA), with 90.1% nt identity and 97.3% aa identity. VP2 was closer to SCZ-3 (MK159152, CHN) and SCZ-6 (MK159153, CHN), with 92.6 and 92.5% nt (85.0 and 84.5% aa) identity, respectively, whereas it had 93.9% aa identity to 42FR (MF784576, Egypt).

**Table 1:** Nucleotide and amino acid identities (%) between CH-HNSC-2018 and other bovine norovirus strains (14 strains: 6 full-length and 8 partial sequences)

Strain	GenBank accession no.	Genome length (nt)	Genome nucleotide identities	Gene^a^							
				p48	NTPase	p22	VPg	Pro	RdRp	VP1	VP2
Newbury2	AF097917	7311	85.2	81.8/92.1	85.3/98.3	82.8/95.1	83.3/95.9	85.6/97.2	86.1/97.8	88.0/96.0	84.2/92.2
Dumfries	AY126474	7311	85.1	82.4/91.2	85.0/98.6	85.7/95.1	81.4/92.6	86.0/97.2	85.7/97.8	86.7/95.8	85.1/92.2
B309	EU794907	7317	85.0	81.8/90.0	85.3/98.1	84.4/95.1	81.1/93.4	85.1/97.2	86.4/97.8	86.2/96.2	86.0/91.2
Adam2006	NC_029645	7313	86.5	84.4/94.2	85.8/97.8	84.4/94.0	86.6/94.3	87.8/96.7	88.9/99.0	86.6/95.4	85.1/91.7
BET-17	MK159169	7321	87.6	95.4/97.3	95.6/98.9	96.6/97.8	96.2/99.2	96.1/98.3	97.0/99.6	67.2/70.6	52.3/57.3
Jena	AJ011099	7338	85.1	72.8/77.6	77.4/92.1	64.4/68.8	68.8/75.7	72.1/87.0	75.2/89.7	67.9/69.2	63.3/68.2
BET-14	MK159175	2683	NA	NA	NA	NA	NA	NA	NA	67.0/70.4	52.4/57.1
BET-1	MK159174	2332	NA	NA	NA	NA	NA	NA	NA	67.2/70.6	NA
MILQ-1	MK159173	2332	NA	NA	NA	NA	NA	NA	NA	67.2/70.6	NA
MIQW-12	MK159172	2332	NA	NA	NA	NA	NA	NA	NA	67.2/70.6	NA
MI-43	MK159171	2332	NA	NA	NA	NA	NA	NA	NA	66.9/70.6	NA
MI-48	MK159170	2332	NA	NA	NA	NA	NA	NA	NA	66.9/70.6	NA
SCZ-3	MK159152	803	NA	NA	NA	NA	NA	NA	NA	NA	92.6/85.0
SCZ-6	MK159153	803	NA	NA	NA	NA	NA	NA	NA	NA	92.5/84.5

NA, the gene not available or the available sequence is too short to compare

^a^ Nucleotide / amino acid identities (%)

**Fig. 1:**
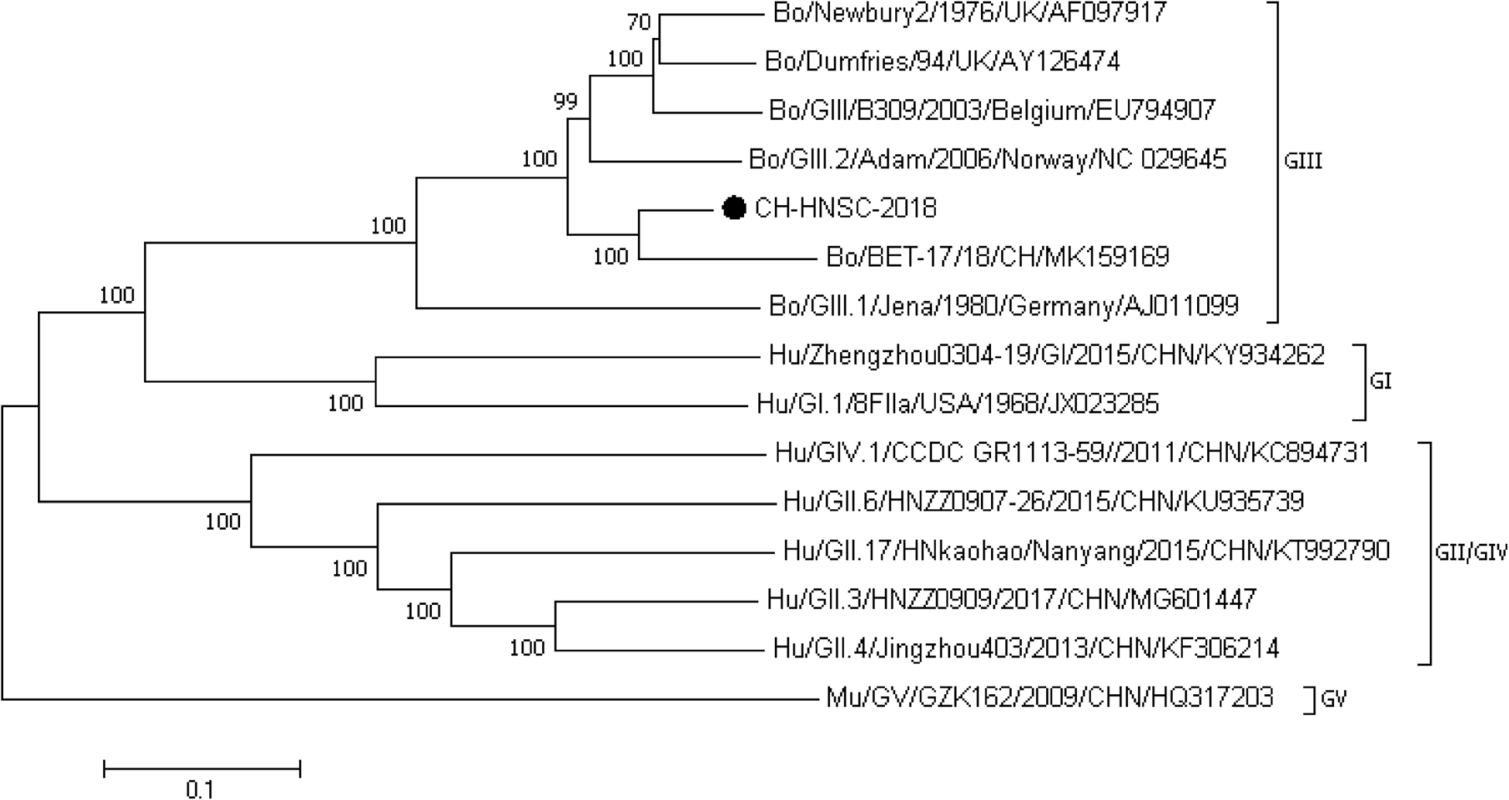
Phylogenetic analyses of the whole genome sequence of CH-HNSC-2018. A phylogenetic tree was generated by MEGA 7.0 software using the neighbour-joining method with 1000 bootstrap replicates

**Fig. 2:**
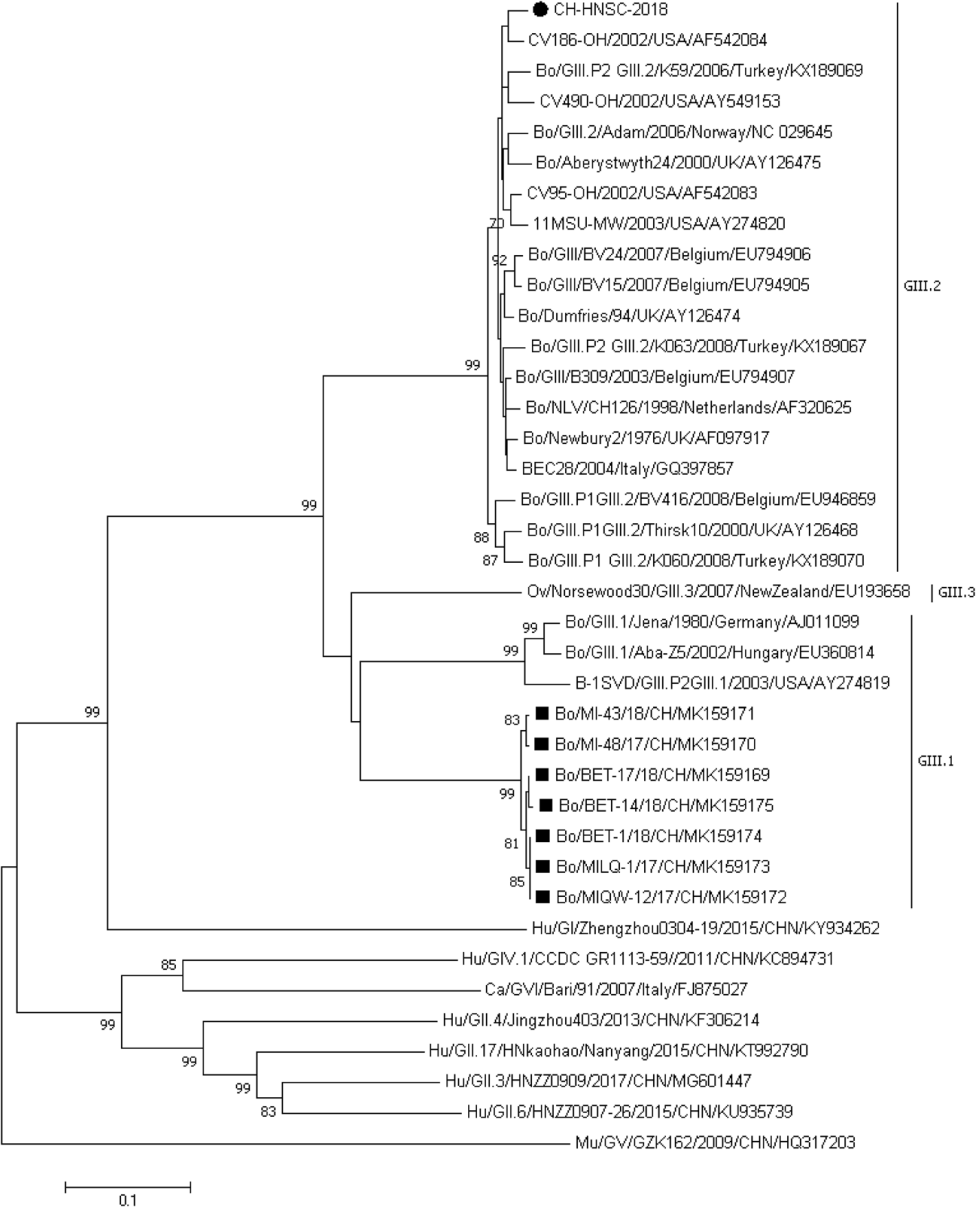
Phylogenetic analyses of VP1 based on the deduced aa sequences. A phylogenetic tree was constructed by MEGA 7.0 software using the neighbour-joining method with 1000 bootstrap replicates. *Circle*, the strain identified in this study; *square*, strains detected in China by other groups

We further generated phylogenetic trees based on the whole genome of our BNoV strain and other previously reported sequences. The results demonstrated that CH-HNSC-2018 was most closely related to GIII.2 BNoV strains (Fig. 1). The BNoV VP1 gene of our strain included an ORF of 1569 nt. These nt sequences encoded predicted proteins containing 523 aa residues. Based on the nt sequence differences of VP1, NoVs could be divided into seven genogroups (GI to GVII), and CH-HNSC-2018 was further confirmed to belong to the GIII 2 group (Fig. 2). This is the first time that a GIII.2 NoV has been detected in the VP1 region in China. Recombination events among BNoV strains were further investigated using RDP4 software, but no correlation was detected. This result showed that no recombination had occurred; however, the available BNoV sequences are limited, so there may not be enough data available to confirm this result. Therefore, further studies are required.

To investigate the aetiologic features of the newly identified BNoV strain, we next analysed aa substitution mutations of CH-HNSC-2018. Compared to the other available BNoV strains in GenBank, CH-HNSC-2018 contains several aa substitutions in the VP1 protein, 19 aa substitutions throughout the P domain, and 11 aa substitutions in the P2 domain. Interestingly, 4 new aa mutations, 134A, 327 T, 380 L and 423A, were identified in the VP1 protein of CH-HNSC-2018. (Additional file 1: Table S1). One epitope (E394D) change was found in these substitutions compared to the Newbury2 strain.

## Discussion

Similar to other regions throughout the world, diarrhoea is the most important cause of disease symptoms in calves in China and leads to serious economic losses of cattle producers. NoV is one of the leading causes of human and animal gastroenteritis; however, BNoV is not usually included in the diagnostic algorithms of calf diarrhoea. In this study, during pathogen detection of diarrhoeal calves, only BNoV was detected, and other enteric viruses, *Coccidium* species and *Cryptosporidium* species were not found. However, the relationship between BNoV infection and diarrhoea in calves requires further epidemiological and experimental studies.

The detection rate of BNoV reported in this study (25.0%) is comparatively higher than that reported in South Korea, 2.8% [20]; Argentina, 3.3% [10]; and Belgium, 7.5% [21]. However, the detection rate of BNoV reported in this study is lower than that reported in other studies: 39.5% in Iran [8], 49.6% in Norway [22] and 72.1% in the USA [12]. Despite these differences, a reliable comparison cannot be made among these frequencies because the samples originated from the same farm in the present study.

The new strain identified in our study was BNoV-GIII 2. Considering the previously reported GIII.1 strains identified in China [7], both genotype GIII.1- and GIII.2-related sequences have been found to circulate in Chinese dairy calves. Due to the limited epidemiological data on BNoV infections in China, the dominant strains cannot be accurately confirmed. More studies on the epidemiology of this emerging pathogen should be carried out in Henan Province and other provinces in China. A BNoV outbreak in China was reported in Hebei and Sichuan Provinces in 2018, and the 3 strains identified were BNoV-GIII.1 based on the partial region of the RdRp polymerase ORF (532 bp) [7]. Nevertheless, the BNoV strain detected in our study was BNoV-GIII 2. Given that strains with different BNoV genotypes co-exist in China, prevention and control are more complex. The results of our study will facilitate further research on the evolution and molecular pathogenesis of BNoV. The importance of BNoV surveillance should be stressed given the cattle industry-intensive area of China.

VP1 is the major structural component of caliciviruses and is involved in receptor recognition, host specificity, strain antigenic diversity, and immunogenicity [23]. X-ray crystallography structures for NoV [24] reveal that the main icosahedral core of the capsid is composed of a conserved S domain and a variable P domain. A sequence comparison and phylogenetic studies were conducted using complete VP1 sequences as phylogenetic markers. It is important to evaluate whether aa changes mainly occur in the P domain or the S domain. The P domain is further divided into a comparatively conserved P1 domain and a highly variable P2 subdomain. The latter has an external localization, and compatible with their functions, both cell receptors are involved in interactions with the host cell membrane [25] and have the most important epitopes [26]. In the present report, we identified four unique aa mutations of VP1 in CH-HNSC-2018 (134A, 327 T, 380 L and 423A) and two (327 T and 380 L) within the P2 subdomain. The hypervariable P2 domain contains putative receptor-binding sites and is responsible for host specificity and strain diversity [23, 24]. Therefore, it was speculated that the large shift in aa in the P2 domain may have major implications for the immunogenic characteristics of this novel genotype as well as significant implications for further vaccine development [27]. These substitutions may have major implications in the immunogenic characteristics of the emerging strain CH-HNSC-2018. Thus, further study of the impact of these aa mutations in the VP1 region on the function of the VP1 protein is required.

Previous studies have shown that the S domain is a conserved region within VP1 and have described the icosahedral scaffold of VP1, and a recent study reported that the S domain of a NoV strain contains strain-specific epitopes that contribute to antigenic diversity [28]. This region in strain CH-HNSC-2018 included a 134A unique aa mutation compared with the other NoV strains. This novel mutation further indicates that the S domain of NoV might possess strain-specific epitopes.

To date, there is no suitable cell culture system for BNoV. The lack of pure viral stocks after culture limits the available information regarding the pathogenesis of BNoV and hampers functional studies of their replication. Although BNoV can infect cattle of different ages, interestingly, in the outbreak investigated in this study, only calves aged less than 1 month were infected. This situation may be linked to the geographical distribution, breeding systems or aa substitutions.

## Conclusions

In conclusion, in this study, we detected GIII.2 for the first time in the VP1 region in a diarrhoeal faecal sample in China and sequenced the complete genome of the strain. The results of our study indicated that BNoV was associated with bovine diarrhoea. BNoVs should be considered in the differential diagnosis of calf diarrhoea and are candidates for inclusion in future vaccines in cattle. Further research needs to be conducted to better understand the epidemiology, pathogenic mechanisms, and potential cross-species transmission of this novel BNoV.

## Methods

In April 2018, an outbreak of calf diarrhoea occurred on a bovine farm in Henan Province. According to the farm workers’ descriptions, the morbidity of calves less than one month old was 100% (56/56), and the mortality was approximately 12.5% (7/56); the affected calves displayed muddy, watery, or bloody diarrheic stool and were anorexic and lethargic. The outbreak lasted for more than 10 days, despite routine treatment with antibiotics, including sulfonamides, penicillin, cephalosporin, and enrofloxacin, which were less efficient. Meanwhile, no clinical signs were observed in other bovine herd age groups, including young and adult cattle, during the outbreak. To diagnose the enteric pathogens, a total of eight faecal samples were collected from sick calves and used for RNA extraction and virus isolation. The faecal samples were also tested for *Coccidium* species and *Cryptosporidium* species using the sucrose floatation method.

Faecal suspensions of each sample were prepared by diluting faeces 1:10 (w/v) in sterile phosphate-buffered saline (pH 7.2). These sample suspensions were mixed for 30 s and centrifuged at 8000 g for 10 min at 4 °C. The supernatants were collected and maintained at − 80 °C until further processing.

Total RNA was extracted from 200 μL of the supernatants using the TaKaRa MiniBEST Viral RNA/DNA Extraction Kit Ver.5.0 (Cat# 9766, TaKaRa Bio Inc.) according to the manufacturer’s instructions, and the RNA was maintained at − 80 °C. Reverse transcription was performed using the PrimeScript II 1st Strand cDNA Synthesis Transcription Kit (Cat# 6210A, TaKaRa Bio Inc.) for first-strand synthesis. All samples were examined for BNoV, BCoV, BRV (A, B, C), BVDV, BKoV, BAstV, BNebV, and BToV, which can cause calf diarrhoea, and were detected by RT-PCR using specific primers (Table 2) as described previously. To confirm the accuracy of the results, all tests were performed three times. The positive samples were then filtered through a 0.22 μm filter and diluted with DMEM containing 1% penicillin and streptomycin. Then, Madin-Darby bovine kidney (MDBK, ATCC) cells were incubated with the dilution to observe the cytopathic effect (CPE) daily. After five blind passages, the whole cell lysates were centrifuged and then analysed for BNoV.

**Table 2:** Primers used for the detection of viruses in faecal samples from diarrheic calves

Virus	Sequence (5^′^-3^′^)	Amplicon size (bp)	References
BNoV	CBECu-F: AGTTAYTTTTCCTTYTAYGGBGA	532	[12]
	CBECu-R: AGTGTCTCTGTCAGTCATCTTCAT		
BCoV	F: GCAATCCAGTAGTAGAGCGT	700	[29]
	R: CTTAGTGGCATCCTTGCCAA		
BRV A	F: GCCTTTAAAAGCGAGAATTT	1060	[30]
	R: GGTCACATCATACAAYTC TA		
BRV B	F: GGAAATAATCAGAGATG	795	[31]
	R: CTACTCGTTTGGCTCCCTCC		
BRV C	F: TCAAGAAATGGWATGCAACC	585	[32]
	R: CATAGCMGCTGGTCTWATCA		
BVDV	F: GCTAGCCATGCCCTTAG	290	[33]
	R: CCATGTGCCATGTACAG		
BKoV	F: TGGAYTACAAGRATGTTTTGATGC	216	[34]
	R: TGTTGTTRATGATGGTGTTGA		
BAstV	F: GAYTGGACBCGHTWTGATGG	432	[35]
	R: KYTTRACCCACATNCCAA		
BNebV	F: CAGCCCGTCTGGGTGAAT	524	[7]
	R: CCAGCGTTAGCGTTCCAG		
BToV	F: TTCTTACTACACTTTTTGGA	603	[32]
	R: ACTCAAACTTAACACTAG AC		

^a^ *F* forward primer for RT-PCR, *R* reverse primer for RT-PCR

The complete genome of BNoV was amplified from eight overlapping fragments with specific primers and the 3’RACE method (Additional file 1: Table S2). Briefly, eight pairs of primers were designed based on the conserved regions of available BNoV strains in the GenBank database. The PCR products were cloned into the pMD18-T vector and sequenced (Sangon, China). We further designed special primers flanking the genome (3 race-F) and then acquired the 3’UTR region by semi-nested PCR according to the instructions of RACE kits (Cat# 18373–019, Invitrogen).

The sequences were assembled with SeqMan and then aligned using MegAlign software (version 7.0; DNASTAR Inc., WI, USA). Pairwise sequence identity calculations were performed using Clustal W software within DNAStar 7.0 software (available at https://www.dnastar.com/). The phylogenetic tree was analysed using MEGA 7.0 software (available at http://www.megasoftware.net/) with the neighbour-joining method with 1,000 bootstrap replicates. Meanwhile, recombination events among BNoV strains were further investigated using RDP4 software with seven different algorithms, RDP, Bootscan, MaxChi, GeneConv, Chimaera, SiScan, and 3Seq [36].

To further characterize the VP1 sequences, an aa alignment was performed using all 29 complete BNoV VP1 sequences available in the GenBank database. The VP1 protein forms an icosahedral particle with two principal domains, a protruding (P) domain and shell (S) domain. The S domain resides between residues 48 and 218. The more variable, surface-exposed P domain is predicted to be located between residues 219 and 511, with the P1 domain mapped from 219 to 270 and from 397 to 511 and the P2 domain mapped from 271 to 396 of the capsid.

## Additional files

Additional file 1: **Table S1.** Amino acid substitution mutations of VP1 in CH-HNSC-2018 and other BNoV strains worldwide. **Table S2.** Primers used for complete genome sequencing (DOCX 23 kb)

Additional file 2: **Figure S1.** Schematic genomic organization of CH-HNSC-2018. Each polyprotein gene is indicated in a box. The nt position of each gene is shown on each gene-box border. The predicted protease cleavage sites are shown below the gene boxes. The figures on either side of the conserved norovirus aa motifs, shown below the gene boxes, are the locations in CH-HNSC-2018. (TIF 803 kb)

## Data Availability

All data generated or analysed during this study are included in the published article and its additional files, and the dataset analysed in the current study is available from the corresponding author upon reasonable request.

## Abbreviations

BAstV: Bovine astrovirus
BCoV: Bovine coronavirus
BKV: Bovine kobuvirus
BNoV: Bovine norovirus
BRV: Bovine rotavirus
BToV: Bovine torovirus
BVDV: Bovine viral diarrhoea virus
NoV: Norovirus
