# Supplementary material for: Genetic and phylogenetic analyses of the first GIII.2 bovine norovirus in China
Source: BMC Vet Res. 2019 Sep 2;15:311. doi: 10.1186/s12917-019-2060-0 (PMC6720400; doi:10.1186/s12917-019-2060-0)
Supplement: Supplementary file 1 — Table S1. Amino acid substitution mutations of VP1 in CH-HNSC-2018 and other BNoV strains worldwide. Table S2. Primers used for complete genome sequencing (DOCX 23 kb) [file 12917_2019_2060_MOESM1_ESM.docx]

**TABLE S1** Amino acid substitution mutations of VP1 in CH-HNSC-2018 and other BNoV strains worldwide (29 strains)

| Strain  Position | HNSC | Geographic location | | | | Location  (protein) |
| --- | --- | --- | --- | --- | --- | --- |
|  |  | China (7) | UK (4) | USA (5) | European continent (13) |  |
| 134 | A^‡^ | T | T | T | T | S |
| 164 | V | I | I | I/V | I/V |  |
| 222 | T | I/T | P/V | P/T | A/P/T/V | P1 |
| 240 | M | P | V/M | M | M/P/L |  |
| 251 | N | T | S/N | N | N/S/A/T |  |
| 278 | A | G | S/A | A/G | S/A/G |  |
| 287 | R | A | I | R | I/R/V | P2 |
| 327 | T^‡^ | L | V | V/M | V/L |  |
| 330 | N | G | S | S/N | S/D |  |
| 335 | Q | Y | G | G/E/W/Q | G/W/E/Y |  |
| 337 | C | R | R | R/C/Q | R/Q |  |
| 344 | L | G | P | P/L/Q | L/P/A |  |
| 368 | R | V | H | H/R | H/R |  |
| 369 | F | L | L/F | F/L | L/F |  |
| 380 | L^‡^ | F | F | F | F |  |
| 389 | D | D | E | E/D | D/E |  |
| 394^§^ | D | E | E/D | E/D | E/D |  |
| 418 | S | E | L | L/S/Q | L/S/Q/H | P1 |
| 423 | A^‡^ | P | P | P | P |  |
| 468 | A | C | C/A | A | A/C |  |
| 510 | T | V | A/T | A/T/L | A/I/L/V |  |

^‡^: Specific amino acid mutation found only in CH-HNSC-2018

^§^: One of the putative conformational epitopes for capsid VP1 proteins

China, MK159169, MK159170, MK159171, MK159172, MK159173, MK159174 and MK159175 (GIII.1); UK, AF097917, AY126474, AY126475, AY126468; USA, AF542083, AF542084, AY549153, AY274819, AY274820; European continent: Belgium, EU794905, EU794906, EU794907, EU946859; Italy, GQ397857; Norway, NC_029645; Germany, AJ011099 (GIII.1); Hungary, EU360814; Turkey, KX189067, KX189069, KX189070; Dutch, AF320625, EU193658 (GIII.3).

**TABLE S2** Primers used for complete genome sequencing

| Primers | Sequences (5’-3’) | Length (bp) |
| --- | --- | --- |
| BNoV-1-F | GTGAATGAAGACTTTGACGA | 1684 |
| BNoV-1684-R | GGGGCAGTGTCYGCAATGGCCTG |  |
| BNoV-1587-F | CGARGCBGTTGACCACTGGGACGG | 873 |
| BNoV-2495-R | GCCCGATGGTASCKRCAGTTRTGGGACTC |  |
| BNoV-2329-F | CTTGCCACCCCCCAGCCCACTGTTG | 1222 |
| BNoV-3550-R | GCGCGACCGACTCCTGGGAGTTTGG |  |
| BNoV-3526-F | CCAAACTCCCAGGAGTCGGTCGCGC | 669 |
| BNoV-4194-R | CTCGATGGCATTCATCCCGATCTTG |  |
| BNoV-4135-F | GCCATGGCYTTTGGSCCCTTCTG | 962 |
| BNoV-5096-R | GGGAGGACYTGBCCRAGSGCCGGAGC |  |
| BNoV-4983-F | CCGCTCCATGTTTGCTTGGATGAG | 862 |
| BNoV-5844-R | GATCACCGGAGAGGGTGCAGCGGC |  |
| BNoV-5786-F | CCSATAGCCCARATGATGAG | 832 |
| BNoV-6617-R | AAGARYTCAGAAGCCATCAAG |  |
| BNoV-6488-F | GTCAACCTTGGCGCCTCGGACCAGG | 731 |
| BNoV-7218-R | CAATGGCATCCGRTCTGTATTAAACC |  |
| 3 race-F | GTCAACCTTGGCGCCTCGGACCAGG |  |
| 3 race AUP1 | GGCCACGCGTCGACTAGTACTTTTTTTTTTTTTTTTTT |  |
| 3 race AUP2 | GGCCACGCGTCGACTAGTAC |  |
