# Supplementary figures and images for: Genetic and phylogenetic analyses of the first GIII.2 bovine norovirus in China
Source: BMC Vet Res. 2019 Sep 2;15:311. doi: 10.1186/s12917-019-2060-0 (PMC6720400; doi:10.1186/s12917-019-2060-0)

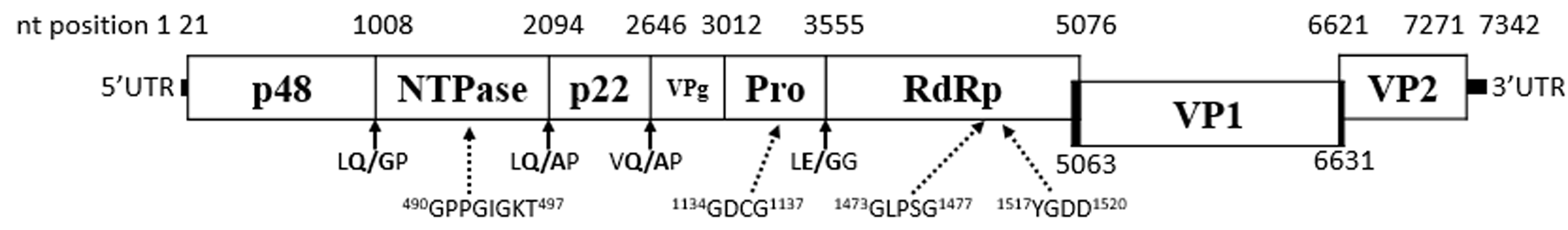

Supplement: Supplementary file 2 — Figure S1. Schematic genomic organization of CH-HNSC-2018. Each polyprotein gene is indicated in a box. The nt position of each gene is shown on each gene-box border. The predicted protease cleavage sites are shown below the gene boxes. The figures on either side of the conserved norovirus aa motifs, shown below the gene boxes, are the locations in CH-HNSC-2018. (TIF 803 kb) [file 12917_2019_2060_MOESM2_ESM.tif]
